# Emerging Roles of Adenosine Metabolism in Astrocytes During Brain Injury

**DOI:** 10.1002/cns.70889

**Published:** 2026-05-15

**Authors:** Shu Zhu, Shanshan Zhong, Xiaoru Lin, Chuansheng Zhao, Yugang Li

**Affiliations:** ^1^ Department of Rehabilitation Shengjing Hospital of China Medical University Shenyang Liaoning Province China; ^2^ Department of Neurology The First Hospital of China Medical University Shenyang Liaoning China; ^3^ Liaoning Provincial Key Laboratory of Big Data for Neurological Diseases Shenyang Liaoning China; ^4^ Department of Neurology, Stroke Center The First Hospital of China Medical University Shenyang Liaoning China

## Abstract

**Objective:**

Adenosine is a key metabolic and neuroregulatory factor in the brain, and an adenosine‐rich immunosuppressive microenvironment is formed post‐stroke, making the adenosine pathway a crucial therapeutic target for improving stroke immunotherapy efficacy. This study aims to address the knowledge gaps hindering adenosine therapy translation, summarize the integrated network of extracellular and intracellular adenosine metabolism, and highlight the dynamic changes of adenosine metabolism in astrocytes and the potential of purine‐converting enzymes as therapeutic targets for cerebral ischemic stroke.

**Methods:**

We conducted a comprehensive summary and analysis of existing research progress over the past two decades, focusing on adenosine metabolic networks (extracellular and intracellular), adenosine metabolic enzymes, subcellular compartmental metabolic pathways, dynamic changes of adenosine metabolism in astrocytes during brain injury, and the role of purine‐converting enzymes in cerebral ischemic stroke. We also reviewed the limitations of current adenosine‐related therapies (e.g., P2Y12‐targeted drugs) and existing knowledge gaps.

**Results:**

Post‐stroke, dying and stressed neuronal cells increase ATP release, which is converted to adenosine by extracellular enzymes, forming an adenosine‐rich immunosuppressive microenvironment. P2Y receptors (a type of ADP receptor) have been extensively studied as vital drug targets for ischemic stroke, but treatments such as Ticagrelor (targeting P2Y12) are associated with severe bleeding. Key knowledge gaps include the lack of cell type‐specific regulation of the adenosine pathway in the brain and insufficient consideration of cell compartmentalized adenosine metabolism. Additionally, astrocyte adenosine metabolism undergoes dynamic changes during brain injury, and purine‐converting enzymes exhibit potential as novel therapeutic targets for cerebral ischemic stroke.

**Conclusions:**

Adenosine metabolism forms an integrated complex network involving extracellular and intracellular processes, with distinct metabolic enzymes and subcellular compartmental pathways. Dynamic changes of adenosine metabolism in astrocytes and purine‐converting enzymes are critical for the development of adenosine‐based therapies for cerebral ischemic stroke. Addressing existing knowledge gaps (e.g., cell type‐specific regulation and compartmentalized metabolism) is essential to overcome current clinical trial difficulties and promote the translation of adenosine therapy for stroke.

## Graphical abstract

Adenosine, as a key metabolic and neurotransmitter in the brain, has become an important therapeutic target for improving the efficacy of immunotherapy for stroke. However, gaps in knowledge about its metabolic pathways have become a key factor limiting its clinical translation. Given current clinical trial difficulties, we summarize the overall view of extracellular and intracellular adenosine metabolism as an integrated complex network and summarize adenosine metabolic enzymes and subcellular compartmental adenosine metabolic pathways. Special attention is paid to the dynamic changes of adenosine metabolism in astrocytes during brain injury and the novel role of purine‐converting enzyme as a therapeutic target for cerebral ischemic stroke.

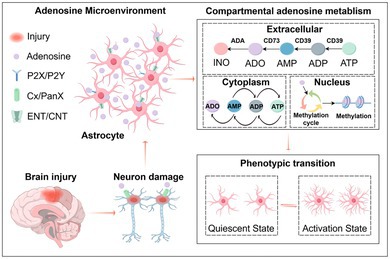

## 1 Introduction

The reparative processes in cerebral regions following cerebral infarction are initiated by a meticulously orchestrated and intricate sequence of events. This process begins with neuronal injury, which triggers a robust inflammatory response (including the release of inflammatory mediators by A1‐reactive astrocytes) that facilitates the degradation and clearance of damaged cells and extracellular matrix components. The subsequent resolution of inflammation triggers a repair response, which primarily involves the activation of A2‐reactive astrocytes and the formation of a gelatinous scar via secreted extracellular matrix components, particularly chondroitin sulfate [1, 2]. Glial scars can limit the area of injury as a physical and chemical barrier and thus play a protective role. However, persistent glial scarring limits regenerative repair of neuronal cells, leading to irreversible stroke‐induced damage [3, 4]. An imbalance in the duration of stimulation or inhibition of astrocyte activation causes ongoing injury to neural progenitor cells or improper healing, leading to defective glial scar formation, which exacerbates the infarcted region of the brain, potentially enlarges the area of injury, and increases the risk of intracerebral hemorrhage [5, 6, 7]. In addition, astrocytes engage in close interactions with neuronal cells by providing metabolic support, antioxidants, and secretion of neuro‐related factors [8, 9, 10]. Consequently, the precise and timely regulation and resolution of astrocyte activation are critical determinants of the quality of repair and healing following brain injury. Achieving an appropriate physiological balance between these phases is essential for optimal recovery.

Recent studies have shown that adenosine metabolism and its regulatory mechanisms are essential for the formation and maintenance of reactive astrocytes. Alterations in intracellular/extracellular adenosine transfer and adenosine deaminase appear to be critical for the phenotypic transformation of astrocytes [11, 12, 13]. Moreover, emerging evidence suggests that adenosine metabolism plays a crucial role in conserving and restoring brain energy supply, thereby facilitating the regulation of crosstalk and communication between astrocytes and neurons [14]. In this paper, we review the definition of adenosine metabolism in the brain as well as the evidence for the regulation of adenosine metabolism in astrocytes in brain injury states, which may represent a new therapeutic target for the repair of therapeutic brain injuries.

## 2 Extracellular Adenosine Metabolism During Brain Injury

Adenosine metabolism exhibits significant compartmentalization, and its role as an extracellular signaling molecule has been well established across various systems, including neurotransmission in the central nervous system [15, 16], blood regulation [17], vascular remodeling within the circulatory system [18, 19], and renal tubular remodeling within the urinary system [20]. In the context of ischemic brain injury, endogenous ATP (iATP) is released into the extracellular space as a result of cellular necrosis, apoptosis, and stress, which are initiated by the disruption of membrane integrity or by changes in mechanical or chemical states that elevate pericellular ATP concentrations [21, 22]. Vesicle‐dependent cytotoxicity or nonvesicle‐dependent active release is mainly regulated by ion channels and nucleotide transport proteins, such as maxi‐anion channels (MACs) and connexin/pannexin half‐channels (Cx/Panx) [23, 24, 25]. Extracellular ATP (eATP) functions as a damage‐associated molecular pattern (DAMPs), facilitating the recruitment and infiltration of immune cells to sites of tissue injury. It is essential for inflammasome activation and sustained release of pro‐inflammatory mediators, including tumor necrosis factor‐alpha (TNFα) and interleukin‐1 beta (IL‐1β) [26, 27]. eATP primarily regulates immune cells through seven ionic P2X and eight metabolic P2Y nucleotide receptors. eATP and adenosine levels significantly increase under acute cellular stress caused by brain damage [28]. The application of a sensitive ATP sensor predicated on GPCR activation, termed GRABATP1.0, provided direct evidence that lipopolysaccharide (LPS) elicits a substantial release of ATP from individual astrocytes within the mouse cerebral cortex [29]. Notably, the primary metabolic fate of eATP involves its conversion to extracellular adenosine via a series of enzymatic reactions, which are crucial for attenuating inflammation and facilitating the transition from the inflammatory to the reparative phase following tissue injury. Thus, to understand the role of adenosine in stroke, it is necessary to understand the entire purinergic metabolic state.

**FIGURE 1:**
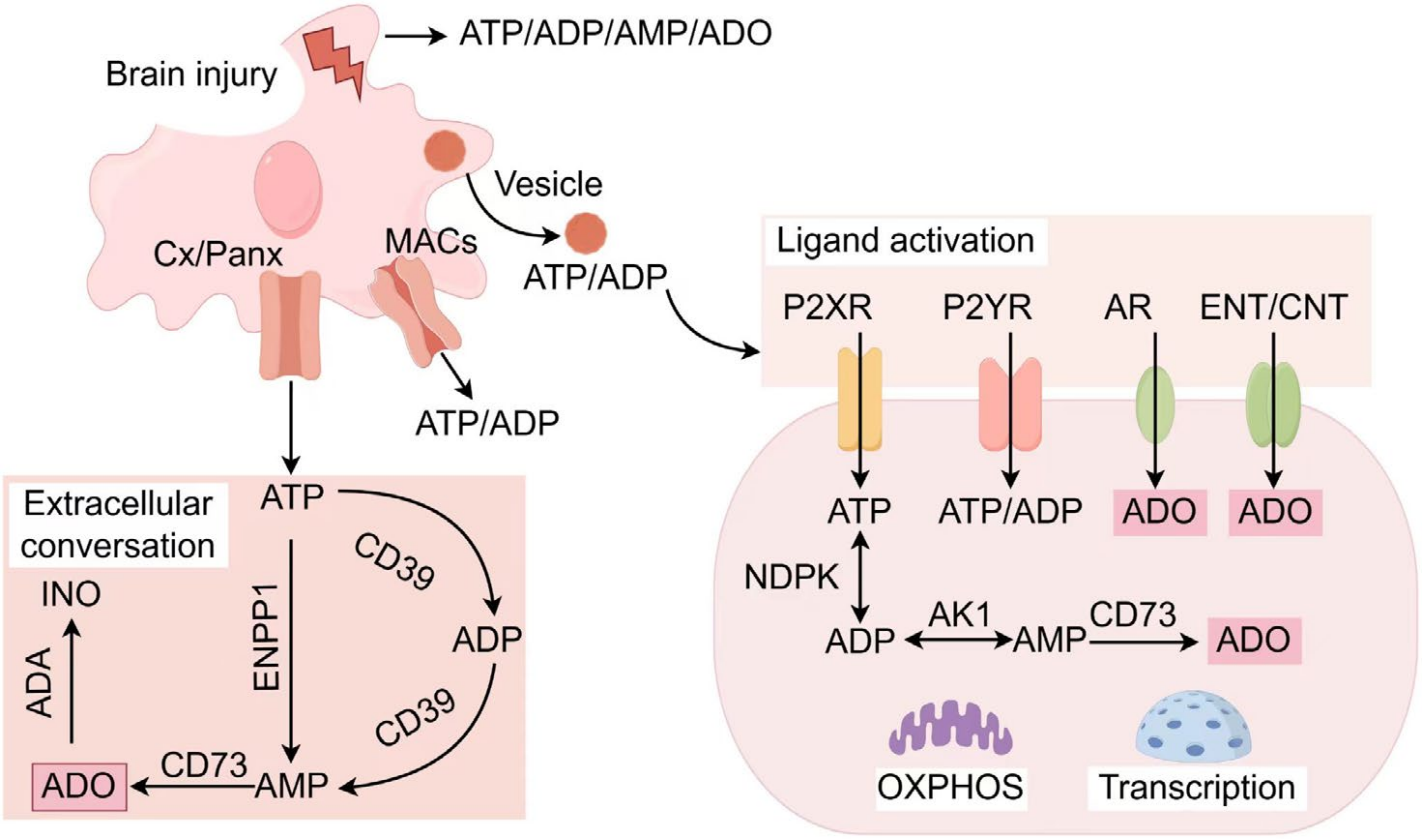
Turnover of adenosinergic signaling pathway. Adenosinergic signaling is composed of several steps. The release of endogenous adenosine or adenosine phosphate after brain injury is mediated by direct release due to cell membrane damage, ion channels and nucleotide transporters: Maxi‐anion channels (MACs), connexin/pannexin half‐channels (Cx/Panx), and vesicle‐dependent exocytosis (Upper left). ATP and ADP signals predominantly via P2X receptors (P2XR) and P2Y receptors (P2YR) on cells within the injury microenvironment. Extracellular adenosine can either be transported into the cell by equilibrative nucleoside transporters (ENTs) and concentrative nucleoside transporter (CNTs) and activate adenosine receptor (AR)‐mediated cellular signaling (Upper right). ATP or adenosine diphosphate ribose (ADPR) can be hydrolyzed directly into AMP by ectonucleotide pyrophosphatase/phosphodiesterase 1 (ENPP1). ATP can also be hydrolyzed to AMP via ectonucleoside triphosphate diphosphohydrolase‐1 (CD39), and AMP is subsequently converted into adenosine by ecto‐5′‐nucleotidase (CD73) (lower right). In addition, intracellular AMP can be further converted into ATP by metabolic enzymes, including adenylate kinase (AK), and nucleoside diphosphate kinase (NDPK) (lower left). This figure was inspired by and conceptually adapted from Yegutkin and Boison [34]. It has been redrawn based on the described methodology and data interpretation to avoid copyright infringement.

The classical adenosine formation pathway involves continuous ATP metabolism via a series of hydrolases, mainly the conversion of eATP to eADP and eAMP, via CD39. Additionally, emerging evidence suggests that ENPP1, which is responsible for the cyclic guanosine monophosphate‐adenosine monophosphate synthase‐stimulator of interferon gene (cGAS‐STING) pathway, directly hydrolyzes eATP to eAMP [30, 31]. This appears to be essential for cells to replenish adenosine pools rapidly in response to tissue damage and repair. Extracellular –5′‐nucleotidase Nt5e (also known as CD73) then performs the final step of converting AMP to adenosine [32]. In addition to the classical eATP‐adenosine hydrolytic conversion route, extracellular adenosine can be converted from CD38‐dependent nicotinamide adenine dinucleotide (NAD), and then ADPR is converted to AMP by ENPP1 to participate in the production of extracellular adenosine [33, 34]. Under the action of extracellular nucleotidase, the extracellular environment transforms from an inflammatory to an anti‐inflammatory environment and transitions from the injury to the repair stage after brain injury. Adenosine has a variety of pathways, including the formation of inosine catalyzed by adenosine deaminase (ADA) and its eventual conversion to hypoxanthine by purine nucleoside phosphorylase (PNP). In addition, adenosine can be taken up and transported into cells via cell membrane nucleoside transporters, including four energy‐independent equilibrative nucleoside transporters (ENT1 to ENT4) and three sodium‐dependent concentrative nucleoside transporters (CNT1, CNT2, and CNT3). Adenosine can also regenerate ATP through adenylate kinase (AK) and nucleoside diphosphate kinase (NDPK) reactions, which provide timely inflammatory production and resolution and extracellular energy supply. The above content is presented visually in Figure 1.

## 3 The Role of Adenosine Metabolic Enzymes in Astrocytes

### 3.1 CD39

CD39, also known as ectonucleoside triphosphate diphosphohydrolase‐1 (ENTPDase‐1), is encoded by the ENTPD1 gene. This transmembrane protein is characterized by two transmembrane domains, a large extracellular hydrophobic domain, and an intracellular domain composed of NH_2_ and COOH‐terminal fragments. The extracellular structural domain encompasses the active center of CD39, hydrolyzing ATP and ADP in a Ca^2+^‐ and Mg^2+^‐ dependent manner [35, 36]. Additionally, it has been demonstrated that CD39 can exist in a soluble catalytically active form in the circulation of both humans and mice [37]. CD39 is expressed in various immune and nonimmune cells, including endothelial cells and fibroblasts. It has been shown to be associated with several mouse ischemia models, including brain [38, 39], kidney [40, 41], myocardium [42, 43], and intestine [44, 45]. Beyond its potential role in converting ATP and ADP to adenosine, CD39 in vascular cells has been reported to diminish platelet activation and recruitment by metabolizing ATP and ADP released by platelets [46]. In recent years, numerous studies have highlighted the role of CD39 in the progression of brain injury. Knocking out CD39 in mice by replacing exons 4–6 (apyase‐conserved regions 2–4) with a PGKneo cassette exhibited an increased cerebral infarct volume. Conversely, supplementation with recombinant soluble human CD39 restored post‐ischemic cerebral perfusion, thereby alleviating the severity of brain injury [38, 47]. In addition, systemic overexpression of CD39, or its overexpression specifically in myeloid cells, resulted in reduced neurological impairment following cerebral ischemia [48]. However, an alternative study demonstrated that the overexpression of CD39 enhances the hyperexcitability of ventral hippocampal pyramidal neurons, potentially increasing the risk of anxiety and depression [49]. Consequently, interventional therapies targeting CD39 in specific cell types within the post‐ischemic brain could be beneficial in limiting infarction while minimizing the likelihood of adverse effects. A recent study found that increasing CD39 expression on the surface of astrocytes through Mdivi‐1 pretreatment limited the extent of ischemic brain damage [50]. Furthermore, defects in ShcC/N‐Shc(Rai) within astrocytes enhanced CD39 expression and activity, which polarized astrocytes toward a neuroprotective phenotype [51]. Therefore, targeting astrocytes to deliver compounds with CD39‐like hydrolase activity may represent a promising strategy.

### 3.2 CD73

CD73, also known as extracellular 5′‐nucleotidase and encoded by the NT5E gene, is a multifunctional transmembrane glycoprotein located on the cell surface. It catalyzes the conversion of extracellular adenosine from AMP [52, 53]. CD73 is anchored to the plasma membrane by glycosylphosphatidylinositol (GPI) and plays a crucial role in the functional regulation of astrocytes as a key enzyme in the production of extracellular adenosine. CD73 consists of a C‐terminal structural domain (which contains the substrate binding site and noncovalently associates with the plasma membrane via a glycosylphosphatidylinositol (GPI) anchor) and an N‐terminal structural domain (which is involved in maintaining binding to zinc ions) and is linked by a short intervening helix that primarily regulates its movement. The attainment of catalytic activity by CD73 requires a transition from a closed to an open conformation, which exposes the active binding site and facilitates the subsequent binding of AMP [54]. CD73 is widely expressed on the cell surfaces of various organisms, including hepatocytes [55, 56], fibroblasts [57, 58, 59], endothelial cells [60, 61], lymphocytes [62, 63], and proximal tubular epithelial cells. Recent single‐cell sequencing of the human brain has revealed that CD73 is abundantly expressed in astrocytes and oligodendrocyte precursor cells. Furthermore, CD73 is prominently present in astrocytes that exhibit strong positivity for hypoxia‐responsive genes such as HILPDA, suggesting that hypoxia may serve as an additional driver of CD73 expression, particularly in conditions of inadequate cerebral blood supply [64]. During the progression of inflammation in the brain, reactive astrocytes significantly upregulate CD73 and express various CD73 variants. These cells are predominantly concentrated in areas of demyelination, leading to the hypothesis that CD73 in astrocytes plays a crucial role in the restoration of myelin damage and the re‐establishment of neuronal signaling following brain injury [65]. Furthermore, pharmacological inhibition of CD73 in astrocytes through various modalities resulted in differential modulation of cell behavior. Specifically, the inhibition of CD73's catalytic activity by the addition of α,β‐methylene ADP (APCP) did not directly impact the migratory capacity of astrocytes; however, it did reduce cell proliferation and alter the expression levels of the A1R receptor. This alteration was reversed by adenosine supplementation, highlighting the necessity of a CD73‐mediated extracellular adenosine pool for the recruitment of astrocytes to sites of injury in response to ischemia [66]. Interestingly, the treatment of astrocytes with polyclonal anti‐CD73 antibodies or CD73 small interfering RNA increased the rate of migration in a scratch wound assay [66]. This finding is supported by a previous study that demonstrated that the knockdown of CD73 in breast cancer cells effectively prevented cell adhesion to the extracellular matrix (ECM) [67]. This suggests that CD73 may have a redundant role independent of adenosine production, potentially serving as a bridge between the ECM and intracellular signaling events, thereby influencing the proliferative and migratory states of astrocytes.

### 3.3 CD38

**FIGURE 2:**
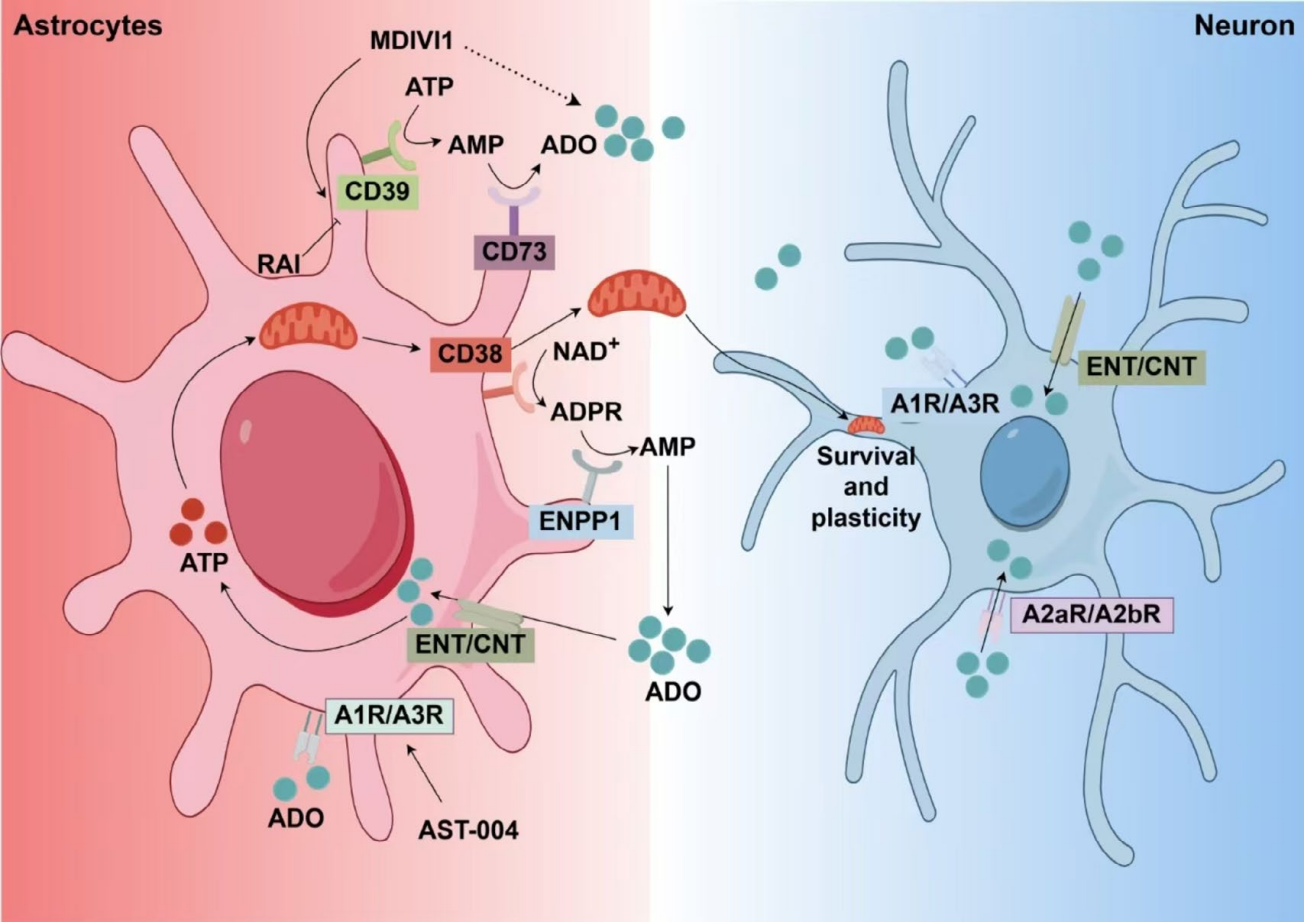
Dependent action of adenosine‐associated receptors in astrocytes and neuron cells. Extracellular adenosine is generated in a stepwise process. Adenosine is transported into and out of the cell by ENT/CNT and signals via AR on neuron cells, which can also re‐enter the astrocytes or activate A1R/A3R on the surface. Intracellular adenosine can be reconverted into ATP to regulate mitochondrial state, and astrocytic release of extracellular mitochondrial into neuronal cells is mediated by ADP‐ribosyl cyclase 1 (CD38). Several approaches have been developed to target adenosine‐associated metabolic enzymes and adenosine receptors on the surface of astrocytes, including enhancing CD39‐mediated establishment of extracellular adenosine microenvironments (supplementation of MDIVI1) and promoting A1R/A3R activation in response to injury (administration of AST‐004).

CD38 is a type II transmembrane glycoprotein characterized by a short N‐terminal cytoplasmic tail, a transmembrane structural domain, and a C‐terminal region located in the extracellular compartment. CD38 utilizes NAD^+^ as a substrate to produce cyclic ADP‐ribose (cADPR), which ultimately contributes to the generation of AMP for adenosine production in the presence of ENPP1 [68, 69]. Research has shown that CD38 is highly expressed during brain development, particularly in astrocytes, and that a deficiency in CD38 leads to impaired development of both astrocytes and oligodendrocytes in mice [70]. Initial studies have shown that astrocytes can directly transfer functional mitochondria to damaged neurons through “Tunneling Nanotubes” (TNTs), thereby increasing their ATP levels and significantly improving neurological function. This process relies on the CD38‐mediated NAD^+^ signaling pathway and the mitochondrial motility protein Miro1. Interference with CD38 signal transduction inhibits the transfer of mitochondria from astrocytes to neurons in a mouse model of focal cerebral ischemia. It was accompanied by a significant reduction of the neural plasticity marker GAP43 in the infarcted area and progressive deterioration of the nervous system [71]. It is worth noting that Microglia, oligodendrocytes and pericytes are activated after ischemic stroke. And whether other glial cells are involved in mitochondrial transfer and their interaction with neurons needs to be further evaluated. Further studies have shown that α‐Synuclein (α‐Syn) aggregates increase the global TV‐mediated connectivity between astrocytes and neuronal cells [72]. However, the decrease of MFN2 in astrocytes contributes to the age‐associated reduction of mitochondrial transfer efficiency and thus compromises the integrity of the blood–brain barrier. In addition, astrocytic low‐density lipoprotein receptor‐related protein‐1 (LRP1) promotes astrocyte‐to‐neuron mitochondria transfer by reducing lactate production and ADP‐ribosylation factor 1 (ARF1) lactylation. Suppression of astrocytic LRP1 reduced mitochondria transfer into damaged neurons and worsened ischemia–reperfusion injury in a mouse model of ischemic stroke [73]. The administration of the monomer component ginsenoside Rb1 can promote the transfer of mitochondria in astrocytes to neurons, while the intracranial knockout of CD38 limits the neuroprotective effect of ginsenoside Rb1 [6]. Disruption of CD38 signaling inhibits mitochondrial transfer from astrocytes to neurons in a mouse model of focal cerebral ischemia. This interference is associated with a significant reduction in the neuroplasticity marker GAP43 in the infarcted area, leading to progressive neurological deterioration. In addition, CD38 signaling plays a crucial role in mediating metabolic and mitochondrial stress in reactive astrocytes. Inflammation‐induced metabolic and mitochondrial stress can be addressed by regulating CD38 cyclase/hydrolase activity. Following TNFα stimulation, CD38 expression in astrocytes significantly increased, which was accompanied by a decreased NAD^+^/NADH ratio and increased mitochondrial fragmentation, resulting in a distinct reactive astrocyte phenotype [74]. However, another study indicated that excessive CD38 expression in reactive astrocytes promotes pro‐inflammatory transcriptional reprogramming and enhances the chemotactic potential of inflammatory monocytes [75]. Therefore, the beneficial effects of CD38 signaling in in vivo astrocytes and the detrimental effects of CD38 signaling in immune cells must be carefully evaluated. Furthermore, since CD38 regulates both intracellular and extracellular NAD^+^, the impact of CD38 activity in astrocytes on pathological aspects following brain injury needs to be further assessed by examining the ratio of extracellular to intracellular NAD+. Figure 2 shows dependent action of adenosine‐associated receptors in astrocytes and neuron cells.

## 4 Regulation of Extracellular Adenosine Transport in Astrocytes Involved in Brain Injury Progression

Extracellular adenosine can enter cells through two types of nucleoside transporters: the equilibrative nucleoside transporters (ENT) from the SLC29 family and the concentrative nucleoside transporters (CNT) from the SLC28 family. Additionally, adenosine can influence cellular behavior by activating adenosine receptors located on the cell surface.

### 4.1 ENT and CNT

CNT is an evolutionarily conserved isotropic transporter protein composed of three isoforms of sodium‐dependent condensed nucleoside transporter proteins: CNT1, CNT2, and CNT3. In contrast, ENT is a sodium‐independent unidirectional transporter protein that includes four classes of typical family members: ENT1 through ENT4. The individual isoforms of ENT are widely expressed throughout the organism, albeit with varying abundance in different tissues. Both ENT and CNT play crucial roles in the nucleotide salvage synthesis pathway, as well as in the uptake of extracellular adenosine and the efflux of intracellular adenosine. ENT and CNT are also responsible for the cellular uptake of nucleoside analogs in various disease states [76, 77]. Moreover, ENT and CNT are essential for the regulation of astrocytes. Astrocytes are particularly adept at nucleoside metabolism and nonmetabolism‐dependent uptake [78]. ENT has been demonstrated to facilitate the uptake of adenosine, which is crucial for the regeneration of ATP by astrocytes, which is vital for the management of brain injury [79]. For example, SIN‐1 (3‐morpholinosydnonimine)‐induced oxidative stress resulted in astrocytes, without affecting its function. This impairment resulted in elevated extracellular adenosine levels in the brain [80]. In addition, impaired transport of elevated extracellular levels of adenosine and ENT1 was demonstrated in isolated primary astrocytes cultured under conditions of glucose and oxygen deprivation [81]. This may reflect a compensatory protective mechanism in the brain during acute stress, which helps prevent sustained and expanding inflammatory damage. Notably, recent studies have demonstrated that the inhibition of ENT1 by small molecules prevents spatial memory decline in mice with Alzheimer's disease [82]. This suggests that the controlled restriction of adenosine uptake in astrocytes is beneficial for protecting against initial brain injury and for maintaining neuronal plasticity.

## 5 Adenosine Receptor‐Dependent Adenosine Action in Astrocytes

Following an ischemic stroke, elevated extracellular adenosine concentrations influence both neuroprotective and neurodegenerative outcomes through four G protein‐coupled adenosine receptors: A1R, A2aR, A2bR, and A3R. However, current research on these receptors presents conflicting findings. For example, both the activation and inhibition of the A2bR receptor have been reported to reduce the progression of brain damage [83, 84]. The development of therapies utilizing high‐affinity adenosine receptor agonists to prevent sustained brain damage remains limited due to the desensitization effects mediated by adenosine and the sustained activation of adenosine receptors, which can trigger peripheral cardiovascular side effects [85]. Throughout the progression of LPS‐induced systemic inflammation, there is a rapid increase in plasma adenosine levels. This elevation subsequently enhances central extracellular adenosine tone and promotes the expression of inflammatory mediators, such as CCL2, CCL5, and CXCL1, in astrocytes via A1Rs [13]. This finding is consistent with previous research demonstrating that astrocytes respond quickly to inflammatory stimuli within hours,undergoing dynamic transcriptomic changes dependent on adenosine binding to A1Rs [86]. A recent study demonstrated a significant reduction in both the rate of lesion growth and the volume of the lesion area through continuous infusion of the A1R/A3R agonist AST‐004 for 22 h, administered 2 h after transient middle cerebral artery occlusion in a nonhuman primate model [87]. This finding suggests that early intervention with A1R/A3R receptor activation during ischemic stroke is effective, potentially due to the creation of a reparative microenvironment facilitated by the rapid clearance of damaged cells, thereby promoting repair. However, further investigation is needed to determine whether the neuroprotective effects of A1R/A3R activation result from enhanced extracellular adenosine clearance or from the modulation of the relative activity of A2aR and A2bR.

Early studies indicated that A2aR in astrocytes plays a crucial role in memory regulation [88]. In recent years, an increasing number of studies have demonstrated that A2aR in astrocytes is essential for the transition between pro‐inflammatory and anti‐inflammatory microenvironments following brain injury. For instance, the activation of the A2aR decreases the release of inflammatory factors mediated by the STAT3/YKL‐40 axis in astrocytes, thereby mitigating white matter damage [89]. Furthermore, the inhibition of A2aR in mice with ischemic stroke resulted in widespread neuronal death and sustained proliferation of type A1‐reactive astrocytes [90]. Previous studies have confirmed that persistent type A1‐reactive astrocytes typically increase brain inflammation, which is detrimental to neurons [91]. Thus, A2aR plays a critical role in the transition from inflammatory abatement to a reparative phenotype following brain injury. There is a relative paucity of studies on A2bR in astrocytes, as adenosine is less potent compared to other adenosine receptors. Previous research has demonstrated that chronic continuous infusion of the A2bR agonist BAY60‐6583 improved focal cerebral ischemic injury and mitigated the extent of ischemia‐induced neuronal death [92]. Notably, a recent study demonstrated that the activation of A2bR on astrocytes recruits the cAMP‐PKA signaling pathway, leading to the rapid activation of glucose metabolism and the release of lactate. This process subsequently replenishes the pool of readily available extracellular energy substrates, which is critical for maintaining synaptic function, particularly during periods of high energy demand or reduced energy supply [14]. These findings suggest that a therapeutic approach aimed at rescuing and preserving brain energy through astrocyte A2bR could be beneficial in stroke treatment, as the site of injury is inherently ischemic and hypoxic. Therefore, enhancing A2bR activity in astrocytes presents a promising therapeutic avenue for prolonging the critical healing period following cerebral infarction.

## 6 Intracellular Adenosine Metabolism in Astrocytes

### 6.1 ADK (Adenosine Kinase)

Intracellularly, adenosine kinase (ADK) serves as a crucial regulator of adenosine and catalyzes the conversion of adenosine to adenosine 5′‐monophosphate [93, 94]. There are two isoforms of ADK: the long isoform (ADK‐L), which is located in the nucleus, and the short isoform (ADK‐S), which is found in the cytoplasm. ADK is predominantly present in neurons during early development, but its abundance increases in astrocytes as development progresses. Initial investigations identified ADK levels in astrocytes as potential predictors and regulators of epileptogenesis [95, 96]. More recent research has demonstrated that ADK in astrocytes also plays a role in the onset and progression of stroke. In a model of focal cerebral ischemia induced by middle cerebral artery occlusion, localized downregulation of ADK was found to mitigate ischemic neuronal damage in the cortex [97]. In addition, in cases of focal brain injury in rats, the expression of ADK was upregulated, leading to the secretion of pro‐inflammatory cytokines by astrocytes. The reduction of the inflammatory microenvironment was facilitated by adenovirus‐mediated knockdown of ADK [98]. However, it is important to note that while in vivo targeting of ADK knockdown in astrocytes suggests a promising neuroprotective strategy, the side effects of gene therapy render it unsuitable as a prophylactic treatment to prevent the recurrence of cerebral ischemia. Therefore, it is prudent to explore less invasive methods to elevate adenosine levels in patients' brains. For instance, the delivery of an antisense oligonucleotide (ADK^ASO^) targeting ADK to reactive A1 astrocytes successfully reduced ADK levels in the brain and alleviated inflammation [99]. In conclusion, the stabilization of the adenosine pool through the inhibition of adenosine pool stabilization by interfering with ADK in astrocytes may help mitigate the extent of injury following cerebral ischemia. This approach could contribute to the development of ischemia‐tolerant phenotypes in patients at risk for cerebral ischemic injury.

### 6.2 ADA (Adenosine Deaminase)

Adenosine deaminase (ADA) exists in two isoforms, ADA1 and ADA2, which catalyze the irreversible deamination of adenosine and deoxyadenosine to produce inosine and deoxyinosine [100]. A retrospective study involving 7913 cases of acute cerebral infarction revealed a significant inverse correlation between serum ADA concentrations and the incidence of acute cerebral infarction [101]. Additionally, smoking and alcohol consumption were found to significantly lower serum ADA concentrations, which are critical factors in the onset of acute cerebral infarction. Furthermore, loss‐of‐function mutations in ADA2 are linked to early and recurrent strokes and manifest with various vascular inflammatory phenotypes [102]. In pathological conditions, elevated levels of ADA are associated with the regulation of fibroblast growth factor 2 (FGF2), which enhances ADA transcription and activity by increasing MAPK phosphorylation [103]. During an ischemic stroke, the regulation of FGF2 is a critical aspect of the affected brain regions. Therapeutic interventions utilizing transcranial magnetic stimulation to enhance neurological recovery following a stroke have been shown to upregulate brain‐derived neurotrophic factor (BDNF) and FGF2, which may play a role in the ADA response in astrocytes [104]. Furthermore, the targeted intracerebral delivery of specific peptide‐modified bFGF has been shown to enhance recovery from ischemia‐induced neuronal injury and improve motor function [105]. Importantly, the inhibition of ADA in astrocytes leads to increased peripheral neuronal toxicity, while the repletion of inosine enhances glycolytic energy production in astrocytes and supports neuronal survival [106]. These findings suggest a potential strategy for mitigating neuronal damage following cerebral ischemia through a combined approach of inosine supplementation and modulation of ADA levels.

### 6.3 AK (Adenylate Kinase)

Adenylate kinase (AK) continuously monitors intracellular adenine nucleotide homeostasis and facilitates the reversible conversion of AMP, derived from adenosine, to ADP. The elevation of AMP levels mediated by AK enhances adenosine production and activates AMPK, a crucial signaling molecule involved in cellular stress adaptation [107]. Consequently, AK may serve as a pivotal regulator in modulating the intracellular response in astrocytes. Preliminary investigations into AK activity across various regions of the normal canine brain have identified a pattern characterized by elevated activity predominantly in gray matter regions, reduced activity in white matter, and moderate activity in areas composed of mixed tissue types [108]. Furthermore, a study has shown a significant increase in AK activity during the acute phase of supratentorial cerebral ischemic infarction in patients [109]. Recent studies have demonstrated that adenylate kinase 4 can promote the ubiquitin‐dependent degradation of PKM2 by enhancing the interaction between PKM2 and the ubiquitin E3 ligase Parkin [110]. This finding offers a pathway for the targeted regulation of adenosine kinases in astrocytes, as reducing PKM2 levels in these cells effectively mitigates persistent astrocyte activation and the resulting excessive inflammatory microenvironment [111].

### 6.4 NDPK (Nucleoside Diphosphate Kinase)

Nucleoside diphosphate kinases (NDPKs) are a highly conserved group of proteins primarily involved in regulating ADP and ATP homeostasis [112]. However, due to the highly dynamic nature of the transition between ATP and AMP, NDPKs have not been extensively studied in astrocytes. Notably, a previous study analyzing plasma samples from stroke patients demonstrated a significant increase in NDPK‐A levels, indicating its potential as a plasma biomarker for the early diagnosis of stroke [113]. A recent study demonstrated that NDPK‐7 acts as a positive regulator of Wnt/β‐catenin signaling by promoting β‐catenin activation through binding to and phosphorylating the serine residue of glycogen synthase kinase‐3β (GSK3β) [114]. Notably, sustained β‐catenin activation and nuclear translocation play crucial roles in regulating the characteristic changes associated with astrocyte activation [115]. Therefore, it can be hypothesized that NDPK‐7 is essential for mediating the nuclear anti‐inflammatory phenotype of astrocytes following cerebral ischemic injury.

In addition, brain injury can lead to severe mitochondrial dysfunction in nerve cells. The dysfunction of mitochondrial complex functions leads to the decrease in the efficiency of the electron transport chain and the loss of membrane potential, thereby inhibiting the oxidative phosphorylation process, resulting in a significant decrease in ATP synthesis, whereas the intracellular levels of ADP and AMP increase significantly [116, 117]. This change initially appears to be the result of cellular energy depletion. However, during the stage when energy metabolism gradually recovers, the elevated ADP level may instead serve as the dynamic basis for ATP resynthesis, promoting the metabolic repair of nerve cells through the following mechanisms. First, ADP is a key substrate in the catalytic reaction of ATP synthase (Complex V). When the ETC function is partially retained or gradually restored, the electron transfer of NADH and FADH₂ can still drive the proton transmembrane pumping out, forming an electrochemical gradient (Δψm) [118]. The elevated ADP level enhances the substrate availability of ATP synthase, accelerates the conversion process from ADP to ATP, and thereby increases the regeneration rate of ATP in mitochondria [119]. This not only alleviates the energy crisis after ischemia, but also provides support for the maintenance of energy‐dependent functional functions such as membrane pumps (such as Na^+^/K^+^‐ATPase). Secondly, the accumulation of ADP and AMP can activate the AMPK (AMP‐activated protein kinase) pathway, which is the main regulatory factor for cells to sense energy states. The activation of AMPK promotes the upregulation of glucose transporters (GLUTs), enhances glycolysis, and simultaneously induces mitochondrial biogenesis (via PGC‐1α), increased fatty acid oxidation, and autophagy for the clearance of damaged mitochondria (i.e., mitochondrial autophagy) [120, 121]. These effects all contribute to improving the energy metabolism environment of damaged neurons. Furthermore, ADP may also promote the oxidation of NADH to a certain extent through a feedback mechanism, thereby enhancing the overall flux of the electron transport chain [122]. This not only increases the rate of ATP synthesis, but also may reduce oxidative stress and reactive oxygen species (ROS) generation, which helps alleviate secondary neuronal damage. In conclusion, ADP is not merely a metabolic waste in the metabolic recovery stage after brain injury, but rather a key signaling molecule and substrate for energy metabolism repair. We highlighted it in red font in the revised manuscript.

## 7 Intranuclear Adenosine Metabolism in Astrocytes

**FIGURE 3:**
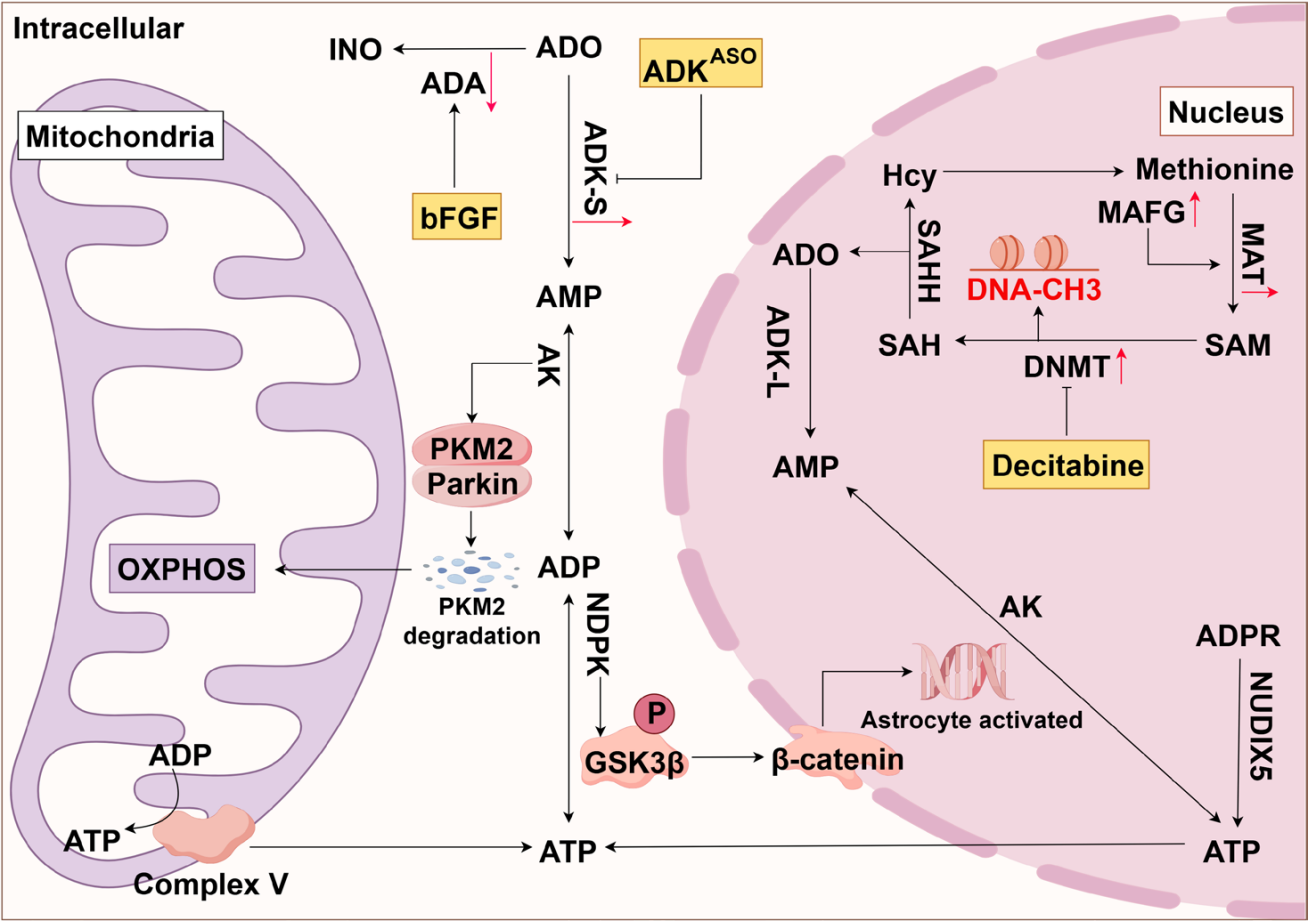
Biochemical compartmentalization of adenosine metabolism in astrocytes. In the cytoplasm, adenosine relies on adenosine deaminase, the cytoplasmic form of adenosine kinase (ADK‐S), AK and NDPK to regenerate ATP. In addition, promoting AK activities contribute to the degradation of pyruvate kinase M2 (PKM2). Enhancing NDPK activities contribute to the enucleation of β‐catenin and activation of astrocytes via phosphorylation of glycogen synthase kinase 3β (GSK3β). In the nucleus, adenosine is associated with transmethylation pathway. DNA methyltransferases (DNMTs) added methyl groups from S‐adenosylmethionine (SAM) to DNA (DNA‐CH3), and SAM is demethylated to S‐Adenosylhomocysteine (SAH). SAH can also be hydrolyzed to adenosine and homocysteine (Hcy) by S‐adenosyl‐L‐homocysteine hydrolase (SAHH). And then SAM is regenerated by methionine adenosyltransferase (MAT). Importantly, the nucleate form of adenosine kinase (ADK‐L) drives the flux of methyl groups by controlling adenosine flux in the nucleus. In addition, ADP‐ribose (ADPR) is used by the pyrophosphatase NUDIX5 to generate nuclear ATP, which is linked to adenosine storage through the conversion of AMP. The red arrow shows changes in enzyme activity in astrocytes during brain injury. The yellow rectangles represent current treatments for adenosine‐related enzymes.

Within the nucleus, adenosine plays a crucial role in the transmethylation process, a biochemical pathway primarily involving DNA methyltransferases that facilitate the transfer of a methyl group from S‐adenosylmethionine (SAM) to DNA, thereby completing the methylation reaction [123]. During this process, SAM is converted into S‐adenosylhomocysteine (SAH). Subsequently, S‐adenosylhomocysteine can be hydrolyzed into L‐homocysteine and adenosine by the S‐adenosylhomocysteine hydrolase (SAHH). Notably, the reaction catalyzed by SAHH is reversible, making the nuclear concentration of adenosine a critical determinant of the extent of DNA methylation [124]. Because adenosine can also be utilized by ADK‐L in the nucleus for conversion to AMP, the activity and expression of ADK‐L in the nucleus indirectly influence methyl flux and the extent of DNA methylation through various biochemical pathways [125]. Transient cerebral ischemia, which leads to oxidative stress or prolonged exposure to inflammatory signals, alters the transcriptional program of astrocytes, thereby mediating their conversion to the A1/A2 phenotype. Notably, changes in the methylation status of astrocytes are a critical feature of the stroke process. For example, astrocytes exposed to LPS transformed into A1‐responsive astrocytes and exhibited sustained DNA hypermethylation, which increased the expression of pro‐inflammatory cytokines such as IL‐1β and IL‐6 [126]. Similar hypermethylation was observed in hyperreactive astrocytes in the penumbra following traumatic brain injury in vivo [127]. Furthermore, supplementation with the DNA methyltransferase (DNMT) inhibitor decitabine was able to reverse persistent inflammatory gene expression [126]. Upon initial exposure to the inflammatory microenvironment, astrocytes exhibit the ability to upregulate MAFG, a member of the small MAF protein family, which is characterized as a basic region and leucine zipper (bZIP)‐type transcription factor. This upregulation enhances the binding to methionine adenosyltransferase II alpha (MATIIα), resulting in DNA hypermethylation and enabling astrocytes to respond effectively to the inflammatory milieu [128]. Conversely, glutamate‐induced increases in global methylation levels within astrocytes contribute to excitotoxicity and play a significant role in the modulation of neuronal damage [129]. Significantly, the analysis of DNA methyltransferase levels during the post‐ischemic phase of cerebral ischemia revealed a substantial decrease in the expression of DNMT1 within astrocytes during the late ischemic phase. This reduction is crucial for creating a reparative microenvironment in the region of brain injury [130]. These studies demonstrate the significance of intranuclear methylation levels in regulating the transformation of type A1/A2 astrocytes. However, a significant number of studies have concentrated on mitigating post‐ischemic brain damage by modulating the activity and expression of DNA methyltransferases and DNA demethylases [131, 132, 133]. In contrast, research on the regulation of adenosine levels remains limited. Furthermore, adenosine has the capacity to regenerate ATP, which is essential for activating astrocytes and replenishing the energy supply of neurons adjacent to the injured area. The above is illustrated in Figure 3.

## 8 Current Advances and Challenges in Stroke‐Targeted Adenosine Metabolism

The significant heterogeneity observed in brain structure necessitates the development of advanced methodologies to investigate the interactions between neuronal cells and neighboring host cells, particularly astrocytes. In conjunction with dissociation‐based single‐cell genomic and metabolomic techniques, an objective assessment of cellular heterogeneity and the spatial organization of astrocytes is essential, with a focus on both adenosine‐associated transcribed genes and adenosine metabolites. A comprehensive study integrating single‐cell data from 70 human and 103 mouse investigations has clarified the utility of brain cell mapping in identifying potential neural progenitors in adults, as well as in revealing microenvironmentally induced variations in microglial cells [134]. It is encouraging that single‐cell nuclear RNA sequencing (snRNA‐seq) can be integrated with a diverse array of sequencing methodologies, particularly the recently introduced single‐cell metabolic regulator profiling (scMEP) approach [135]. This integration has significant implications for elucidating the interactions of adenosine metabolism among various subtypes of post‐stroke astrocytes and between these subtypes and neuronal cells. Moreover, understanding the temporal and spatial complexities of adenosine metabolic networks through the application of emerging innovative methodologies is expected to become a focal point of future research. For instance, many contemporary imaging techniques are limited in their ability to accurately identify the location of cells, their phenotypic changes, and the dynamic nature of cellular transformations within a comprehensive three‐dimensional context [136]. The pathology of stroke is characterized by its complexity and variability. Key areas of investigation include the regulation of the inflammatory phenotype of astrocytes following neuronal injury, the mechanisms by which astrocytes detect changes in the tissue microenvironment and undergo transformations in cellular identity, and the role of adenosine metabolism plasticity in astrocytes across different regions in maintaining cellular identity. These topics represent critical issues that warrant further exploration. In recent years, spatial metabolomics has emerged as a catalyst for innovation in the fields of metabolomics and mass spectrometry. This advancement has enabled the mapping of complex transcriptome regulation and metabolic changes in the brains of individuals with traumatic brain injury, as well as the characterization of the relationship between lipid peroxidation‐related genes and neuronal damage [137]. To further advance the field, future technical challenges must be addressed, including enhancing assay sensitivity, accurately identifying adenosine metabolites, and labeling the cellular origins of metabolic products.

In recent years, the adenosine signaling pathway has emerged as a crucial mechanism in cerebral ischemia and psychiatry regulation. Small‐molecule therapeutic strategies targeting this pathway mainly include: (1) inhibition of adenosine production, such as with CD73 inhibitors (e.g., Oleclumab) or CD39 inhibitors; (2) blockade of adenosine receptors, particularly A2A receptor antagonists like CPI‐444 and AZD4635. In addition, a derivative of the bisindole alkaloid indirubin named indirubin‐3′‐monoxime (I3M) efficiently binds and activates A2aR, which prevents impairment of insulin signaling pathways in lipid‐induced adipocytes without any toxic effects [138]; and (3) modulation of adenosine transporters or metabolic enzymes that indirectly regulate adenosine levels. An effective small‐molecule ADA inhibitor, ZYS‐1, dramatically suppressed prostate cancer growth and metastasis [139]. CNX‐774, a preclinical Bruton tyrosine kinase (BTK) inhibitor, sensitizes inhibit ENT1 in pancreatic cancer [140]. Several agents targeting the adenosine pathway are currently in Phase I/II clinical trials and have shown promise. Nevertheless, challenges remain, including potential systemic side effects, lack of predictive biomarkers, and limited efficacy of monotherapies. Future directions involve developing more selective inhibitors, optimizing targeted delivery systems, and designing personalized immunomodulatory regimens. Overall, small‐molecule targeting of the adenosine pathway represents a promising and innovative approach to immune‐based therapy across various disease contexts. Due to the systemic side effects of local adenosine, adenosine enhancement ideally targeting the lesion is a necessary condition for treatment. However, drug delivery in the focal site has difficulties such as repeated delivery and drug loading. Therefore, cell therapy or gene therapy that locally enhances the adenosine system may be the direction of future research. For example, engineered encapsulated fibroblasts, Encapsulated myoblasts, and stem cells can be engineered to release adenosine by using a lentiviral expression system for anti‐ADK miRNA and release adenosine [141]. In conclusion, targeting the regulation of adenosine levels in the body through cell therapy and gene therapy to combat brain damage holds great therapeutic promise.

In addition, emerging research has also shown that adenosine has functions other than cerebral ischemia, such as psychiatry and sleep regulation. Overexpression of the ADK cytoplasmic isomer in mice is sufficient to alter sleep physiology [142], which suggests that ADK may tune and integrate neurotransmitter pathways involved in sleep–wake regulation by controlling adenosine levels and its receptor‐mediated effects, which provides a key target for future drug development to treat insomnia.

Extracellular ATP and adenosine are also neuromodulators that regulate the function of many neurons in the brain. Dysregulation of extracellular ATP leads to a destabilizing effect on the neural network, as found in the etiopathology of many psychiatric diseases [143]. Abnormally high expression of A2AR in the Lateral septum (LS) induces depression by increasing the activity of A2AR‐positive neurons and inhibiting the activity of peripheral neurons [144]. Therefore, adenosine, as a key messenger regulating brain activity, has important research significance in neural activity and psychiatric diseases. With the in‐depth exploration of molecular mechanisms and the continuous improvement of therapeutic strategies, the regulation of adenosine signaling may bring new breakthroughs in the fields of neuroscience and psychiatry.

## 9 Conclusion

To date, the extensive preclinical data obtained from mouse models have yielded only modest outcomes in early clinical trials for stroke. This discrepancy may, in part, be attributed to biological differences between species, which can lead to inconsistencies across models. Notably, many previous studies have predominantly utilized adult male mice. Future research that incorporates older mice, female mice, or mice with conditions such as hyperlipidemia or hypertension may enhance the generalizability of current findings. In addition, a limited understanding of the mechanisms underlying the transformation of key cell types during a stroke has hindered clinical translation. A substantial body of evidence indicates that precise regulation of adenosine levels is essential for astrocyte function and plasticity, as well as for their interactions with neighboring neurons. Evaluating cell‐to‐cell interactions in the brain and the compartmentalized metabolism of adenosine in astrocytes in vivo presents a significant challenge. The advancement of emerging single‐cell transcriptomics and spatial single‐cell metabolomics is anticipated to greatly enhance our understanding of the regulation of adenosine homeostasis between astrocytes and neurons in the brain. Furthermore, novel genetic tools have proven effective in detecting compartmentalized adenosine levels and their effects on various organelles. These innovative tools, combined with recent findings regarding the dysregulation of adenosine homeostasis during a stroke, will serve as a foundation for further research, and by targeting the adenosine metabolic pathway in astrocytes may prove to be an effective strategy for mitigating infarct expansion during a stroke.

## Funding

The authors have nothing to report.

## Ethics Statement

The authors have nothing to report.

## Consent

The authors have nothing to report.

## Conflicts of Interest

The authors declare no conflicts of interest.

## Data Availability

The authors have nothing to report.
